# Role of Bicarbonates and Mannitol in Rhabdomyolysis: A Comprehensive Review

**DOI:** 10.7759/cureus.9742

**Published:** 2020-08-14

**Authors:** Manoj R Somagutta, Sukrut Pagad, Saijanakan Sridharan, Saruja Nanthakumaran, Ashley A Arnold, Vanessa May, Bilal Haider Malik

**Affiliations:** 1 Department of Research, California Institute of Behavioral Neurosciences & Psychology, Fairfield, USA; 2 Department of Research, California Institute of Behavioural Neurosciences & Psychology, Fairfield, USA; 3 Surgery, California Institute of Behavioural Neurosciences & Psychology, Fairfield, USA; 4 Internal Medicine, California Institute of Behavioral Neurosciences & Psychology, Fairfield, USA

**Keywords:** rhabdomyolysis, bicarbonates, mannitol, crush injury, : acute kidney injury, acute renal injury

## Abstract

Rhabdomyolysis is characterized by rapid muscle breakdown and release of intracellular muscle components into the circulation. Acute renal injury is the most common and fatal complication of rhabdomyolysis. The current literature emphasizes the importance of preventing rhabdomyolysis and finding the benefits of sodium bicarbonates and mannitol in its prevention. A PubMed database search for the keywords "Rhabdomyolysis," "Sodium bicarbonate use in rhabdomyolysis," "Mannitol use in rhabdomyolysis," and a Medical Subject Headings (MeSH) search using the keyword "Rhabdomyolysis; Acute Kidney Injury (Subheading-Prevention and control)" generated 10,005 articles overall. After a thorough application of inclusion/exclusion criteria, 37 relevant studies were selected for this literature study. This analysis demonstrates that aggressive early volume resuscitation with normal saline should continue being the principal focus of therapy, and the use of sodium bicarbonate and mannitol in practical situations is not entirely justified. This article also emphasizes the need for future research on this topic and provides recommendations for future research.

## Introduction and background

Rhabdomyolysis is a syndrome characterized by muscle necrosis and the release of intracellular muscle constituents into the circulation [1]. Bywaters and Beall first reported it in four crush injury victims after excavating them from the rubble during the London bombing in 1941 [2]. They noticed that dark urine and brown pigmented casts similar to hemoglobin were deposited in the distal tubules of the victims, leading to significant renal impairment. The causes of rhabdomyolysis are multifactorial. The most common causes in adults are illicit drugs/toxins, alcohol abuse, medical drugs, trauma (crush injury), genetic defects, seizures, and metabolic disorders [3]. Acute kidney injury (AKI) is the most severe complication of rhabdomyolysis regardless of the etiology. The incidence ranges from 13% to 50%, depending on the clinical setting and diagnosing criteria [4]. In the Meijer et al. study, the mortality of patients who developed AKI was 59% vs. 22% in patients who did not develop AKI [5]. As exemplified by this study, early recognition of rhabdomyolysis and prevention of AKI should be the cornerstone of treatment. 

Many studies have shown that progression to significant renal failure can be avoided via early and aggressive saline infusion [6]. The use of sodium bicarbonate and mannitol to deter the development of AKI in rhabdomyolysis is currently controversial. Some studies propose that patients benefit from sodium bicarbonate and mannitol infusions [7]. However, other recent studies suggest against their use to prevent myoglobinuric renal failure as there is little evidence other than from animal studies, retrospective observational studies, and case series to support their routine use [8,9].

This study's objective is to determine the optimal management and review the effectiveness of sodium bicarbonate and mannitol in the prevention of AKI following rhabdomyolysis. This study also aims to summarise the available evidence on this topic and provide recommendations according to current standards for practical guidelines.

## Review

Methods

Literature was searched in PubMed with parallel strategies based on Medical Subject Headings (MeSH) subheadings and regular keywords for data collection. Table 1 shows regular and MeSH keywords used for the literature search. 

**Table 1:** Regular and MeSH keywords search for literature review MeSH: Medical Subject Headings

Regular keyword- Rhabdomyolysis	
Total results	9692
Results selected	1241
Regular keyword- Sodium bicarbonate use in rhabdomyolysis	
Total results	102
Results selected	23
Regular keyword: Mannitol use in rhabdomyolysis	
Total results	91
Results selected	19
MeSH keyword: Rhabdomyolysis; Acute Kidney Injury (Subheading-Prevention and control)	
Total results	120
Results selected	28

Studies were selected after applying the following inclusion/exclusion criteria.

Inclusion Criteria:

1. Human subjects of all age groups

2. Diagnosis of AKI following rhabdomyolysis

3. Paper published in English with no year restriction 

4. The study types were observational studies, systematic review, literature review, and case-series

5. Full-text papers

Exclusion Criteria:

1. Animal studies

2. Non-English literature

3. Clinical trials, single case reports

Results

Table 2 shows the total number of articles after applying inclusion/exclusion criteria in the following order.

**Table 2:** Total number of articles after applying inclusion/exclusion criteria MeSH: Medical Subject Headings

Regular keyword- Rhabdomyolysis	
Total records	9692
Human	8026
English	6456
Full text	5423
Type of studies	1241
Regular keyword: Sodium bicarbonate use in rhabdomyolysis	
Total results	102
Humans	83
English	72
Full text	59
Study designs	23
Regular keyword: Mannitol use in rhabdomyolysis	
Total results	91
Humans	81
English	67
Full text	50
Study designs	19
MeSH keyword: Rhabdomyolysis; Acute Kidney Injury (Subheading-Prevention and control)	
Total results	120
Humans	78
English	58
Full text	52
Study designs	28

A single PubMed database search generated 10,005 articles overall. After applying the inclusion-exclusion criteria, only 1283 potentially relevant articles remained. After screening the abstracts of these articles, 120 studies were included in the final analyses as they met our study's title and objectives. Out of these, 37 studies were used as they provided full text and pertinent information for this review. 

**Figure 1:**
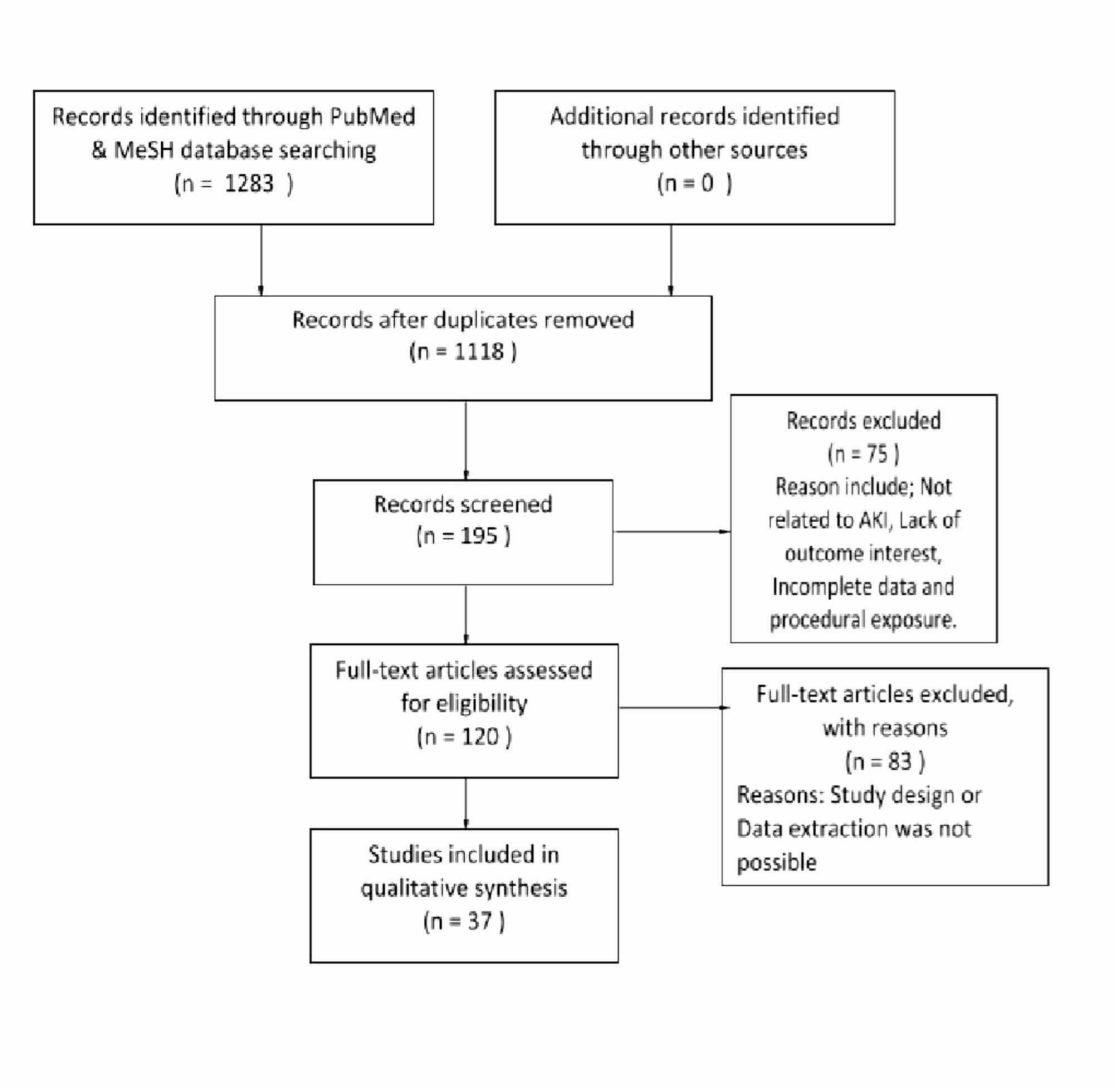
Flow chart explaining the process of current literature review AKI: Acute Kidney Injury, MeSH: Medical Subject Headings

Figure 1 below shows the flowchart with the process of the current literature review.

Discussion 

Etiology

The risk factors for rhabdomyolysis are very diverse. They can be broadly classified into acquired causes and inherited causes. Acquired causes include trauma (crush injury), ischemia, illicit drugs (cocaine, methadone, heroin), alcohol, drugs (statins, fibrates), infections (Epstein-Barr virus, influenza, HIV), extreme temperatures (heatstroke, malignant hyperthermia, malignant neuroleptic syndrome), and toxins (spider bites, wasp stings, snake venom) [10-17]. Some inherited causes such as metabolic disorders (electrolyte changes, diabetic ketoacidosis, thyroid abnormalities) and genetic disorders (disorders of glycolysis or glycogenolysis, lipid metabolism, mitochondrial diseases) are a few of the most common risk factors for rhabdomyolysis [10-17].

Table 3 below summarizes the common causes of rhabdomyolysis. 

**Table 3:** Common causes of rhabdomyolysis

Common causes of rhabdomyolysis
Direct muscular injuries
Excessive exercise
Muscle hypoxia
Ischemic causes
Genetic defects
Metabolic disorders
Endocrine disorders
Electrolyte disorders
Drugs and toxins
Infections and Idiopathic

Complications

Cell destruction promotes leakage of intracellular components causing fatal complications. Immoderate potassium leakage leads to cardiac arrhythmias or cardiac arrest, whereas hypocalcemia is a result of calcium phosphate precipitation with calcification in the necrotic muscle [10-17]. The release of proteases and coagulating cascade from the damaged cell causes hepatic dysfunction and disseminated intravascular coagulation (DIC), respectively. Intracellular fluid accumulation and ischemic changes potentiate the rise in the intra-compartmental pressure and cause compartment syndrome to be an early or late complication [11,14].

Table 4 shows complications of rhabdomyolysis.

**Table 4:** Complications of rhabdomyolysis

Early complications (<12 hrs)	Early or late complications (12-24hrs)	Late complications (>24 hrs)
Hyperkalemia	Compartment syndrome	Acute renal failure
Hypocalcemia		Disseminated Intravascular Coagulation (DIC)
Cardiac arrhythmias		
Cardiac arrest		

Pathogenesis of Myoglobin-Induced Acute Kidney Injury

The exact mechanism of AKI in rhabdomyolysis is complex and debatable with the growing evidence. Although the deposition of myoglobin in the renal tubules remains the main insult, the mechanism by which it occurs remains controversial [18]. 

Myoglobin appears in the urine only when the renal threshold of 0.5 to 1.5 mg of myoglobin per deciliter is exceeded and is grossly visible as reddish-brown (commonly referred to as “tea-colored”) urine when serum myoglobin levels reach 100 mg per deciliter [4]. Acidic urine and incremented uric acid in the urine can further complicate this effect by precipitating myoglobin upon interacting with Tamm-Horsfall protein, resulting in tubular casts formation and obstruction to urine flow [10]. Intratubular myoglobin, when degraded, releases reactive oxygen species and free radicals, causing direct ischemic tubular damage [10]. 

Recent studies demonstrate that in pathological conditions, myoglobin oxidized to ferryl state (Fe) exhibits peroxidase properties and leads to lipid peroxidation. Products of lipid peroxidation contribute to AKI by causing renal vasoconstriction, oxidative injury, and tissue damage [18,19]. The current evidence affirms that kidney failure is due to the collective effects of hypovolemia, aciduria, and direct cytotoxicity due to the accumulation of myoglobin [14,18-20]. Many clinical factors like serum creatine kinase (CK), creatinine, potassium, Ca2+, and urine myoglobin level are valued to foretell the risk of acute renal failure (ARF), but there is no consensus on a single factor [14].

Sodium Bicarbonate

An acidic urine environment potentiates myoglobin-induced renal toxicity [10-14]. The basis behind the use of sodium bicarbonate is that it promotes alkalization of the urine and counteracts the process of heme pigment precipitation, thereby decreasing the direct pigment injury [13-16]. Urine alkalization is also useful in diminishing redox cycling and lipid peroxidation, thus preventing oxidative stress, tubular damage, and renal vasoconstriction [10,17]. Hence, it is believed that urine alkalization, optimizing the pH higher than 6.5, can prevent renal impairment [4]. On the contrary, the evidence is scarce that urine alkalization has a proven clinical benefit over standard saline resuscitation in these patients [6]. 

A well-observed side effect with bicarbonate therapy in the initial stages of treatment is hypocalcemia. It is also noted that when bicarbonates are used in decompensated respiratory patients or circulatory failure, they can cause hyperosmolar states and paradoxical intracellular acidosis [12,21]. However, no studies have compared it as standard therapy with saline resuscitation alone. 

Mannitol

Mannitol is a rapidly acting osmotic diuretic that has many proposed benefits [22]. It works as a renal vasodilator improving glomerular filtration rate. This mechanism leads to diuresis to be beneficial in the excretion of excess myoglobin and the prevention of myoglobin cast formation [22-26]. Mannitol also acts as a free radical scavenger and has an antioxidant effect on renal parenchyma. While some initial studies suggested using mannitol in rhabdomyolysis, most of the evidence presenting mannitol's protective effect comes from animal studies [4]. Further, a few clinical trials and observational studies found no clinical benefit with mannitol [4]. Many studies have observed the paradoxical effect of mannitol as a renal vasoconstrictor when used in higher dosages (>200 g/day), causing osmotic nephrosis [10]. However, many authors support the use of mannitol in rhabdomyolysis-induced renal failure, especially in crush injury victims as mannitol decreases osmotic swelling and edema in the injured muscle cells and helps restore the skeletal muscle function [22,23]. They also recommend that mannitol be administered when saline infusions fail to improve a urine output of more than 300 ml/hr [23], and thus it is practical to initiate treatment with IV fluids and offer mannitol after assessing the urinary response. Frequent monitoring of plasma osmolality and the osmolal gap is needed during mannitol administration, and it should be halted when there is a significant rise in the osmolal gap (>55 mOsm/kg) [10]. 

Table 5 provides a review of the use of sodium bicarbonates and mannitol as adjuvant therapy in rhabdomyolysis.

**Table 5:** Comparative studies on preventive and therapeutic regimens in rhabdomyolysis CPK: Creatine Phosphokinase, ARF: Acute Renal Failure, RP: Rhabdomyolysis Protocol, RM: Rhabdomyolysis, NS: Normal Saline, B: Bicarbonate, M: Mannitol

Title/Author	Study design	Sample size	Patient group	Therapeutic strategy	The outcome in AKI patients
Eneas et al., 1979 [7]	Retrospective	20	Patients with the crush syndrome	Mannitol and sodium bicarbonate	Better in patients with low CPK vs. high CPK
Ron et al., 1984 [27]	Prospective	7	Crush injuries from the collapsed building	Mannitol and Sodium bicarbonate	All recovered without azotemia or renal failure
Knottenbelt et al., 1994 [28]	Retrospective	200	Patients with severe beatings	Fluid loads without mannitol and bicarbonate	No difference; Increased ARF with late admissions
Shimazu et al., 1994 [29]	Retrospective	14	Crush injuries from the earthquake	Early vs Late fluid resuscitation	Better with early and high volume infusions
Homsi et al., 1997 [6]	Retrospective	24	ICU patients	Normal saline vs. normal saline plus bicarbonate and mannitol	No difference
Brown et al., 2004 [30]	Retrospective	2083	Traumatic patients	Normal saline vs. bicarbonate plus mannitol	No difference
Gunal et al., 2004 [31]	Retrospective	16	Patients with crush syndrome	Early vs. late treatment with normal saline followed immediately by bicarbonate	Better with early initiation of treatment.
Cho et al., 2007 [32]	Prospective study	28	Patients with intoxication from doxylamine	Ringer’s lactate vs. normal saline; bicarbonate if urine pH is <6.5	Better if therapy initiated early; Better with a high volume of hydration
Iraj et al., 2011 [33]	Prospective study	638	Earthquake victims with crush injuries.	Early large volume vs Early low volume NS; No sodium bicarb or mannitol associated.	Authors recommend >6 L/day in severe RM and ≥3 L/day IV fluid in moderate RM to decrease the incidence of AKI
Tazmini et al., 2017 [34]	Retrospective	31	Exercise-induced rhabdomyolysis	Normal saline vs. Urinary alkalinization	No significant difference
Nielsen et al., 2017 [35]	Retrospective	77	Traumatic rhabdomyolysis, CPK >10,000 u/l	NS vs NS+B+M(RP)	Reduced ARF was noted with RP. ARF developed in 26% of patients with the RP vs. 70% without it (P= .008).

Many studies have coupled mannitol with bicarbonate to see the synergetic effect, but studies comparing it with saline resuscitation alone are sparse. In 1984, Ron et al. published a study on seven crush victims noticing no visible hemoglobinuria after 48 hrs and no need for hemodialysis in any patients after treating them with sodium bicarbonate and mannitol. A notable impediment of this study was not having a standard control group [27]. According to Scharman et al., sodium bicarbonate and mannitol should be used only to correct metabolic acidosis if present and to achieve a urine output of 300 mL/hr or more, respectively [36]. Brown et al. and Homsi et al. mentioned that the use of bicarbonates and mannitol in patients with rhabdomyolysis seems unrelated or did not improve the clinical outcomes in their study [6,30]. Recently an article published by Michelsen et al. also discouraged the use of bicarbonates and mannitol while categorizing it as a weak recommendation [37]. 

On the contrary, few studies seem favorable to the use of urinary alkalinization and mannitol therapy. Eneas et al. proposed more favorable results in patients treated with mannitol and bicarbonate with low creatine phosphokinase (CPK) than with higher CPK [7]. Gunal et al. recommends that positive results are striking with early vigorous initiation (<12 hrs of insult) and followed by urine alkalization and mannitol therapy [31]. In 2017, Nielsen et al. recognized a significant decrease in the development of acute renal failure of 26% vs. 70% (P=0.008) in patients with CK>10000 u/l with sodium bicarbonate and mannitol added to normal saline compared to patients with normal saline alone, respectively [35].

Why the Need for Revision?

The recent Neilsen et al. study highlights the importance of reassessment on this topic. There is an emerging need to address these topics forthwith, such as a specific clinical biomarker in categorizing rhabdomyolysis and assessing patients for their risk for AKI. The benefit of these agents catered to case specifics such as anuric patients should be evaluated [35]. Also, the appropriate timing for the initiation of therapy and specific parameters for the use of these agents should be determined. There is a demand and need for more multicenter randomized clinical trials to address these issues and provide more evidence. Future research can help practice an evidence-based approach to attain better outcomes and avoid opting for the use of these agents in regular clinical practice. 

Limitations

Nonetheless, the findings of this study have to be seen in light of some limitations. Animal studies and literature published in languages other than English were not used in this review. 

## Conclusions

Rhabdomyolysis is clinically challenging to manage as many medical, social, and environmental factors can contribute to this condition. Acute kidney injury is a fatal insult following rhabdomyolysis, and early identification of the risk factors and interventions to prevention should be the center of treatment for high-risk patients. Considering the historical evidence and theoretical benefits of bicarbonates and mannitol in AKI, it is assumed that these agents are beneficial, which has led to their routine use in standard practice. However, there is ample evidence that these standards of practice need to be revisited. Aggressive early volume resuscitation with normal saline still should be the primary focus of treatment. The use of these agents as a single standard measure is discouraged as they are not superior to saline therapy, as shown by current evidence.
